# Morphometric Patterns and Blood Biochemistry of Capybaras (*Hydrochoerus hydrochaeris*) from Human-Modified Landscapes and Natural Landscapes in Brazil

**DOI:** 10.3390/vetsci8080165

**Published:** 2021-08-13

**Authors:** Hector R. Benatti, Hermes R. Luz, Daniel M. Lima, Vinicius D. Gonçalves, Francisco B. Costa, Vanessa N. Ramos, Daniel M. Aguiar, Richard C. Pacheco, Ubiratan Piovezan, Matias P. J. Szabó, Katia Maria P. M. B. Ferraz, Marcelo B. Labruna

**Affiliations:** 1 Faculdade de Medicina Veterinária e Zootecnia, Universidade de São Paulo, Av Prof. Orlando Marques de Paiva 87, Cidade Universitária, São Paulo 05508-270, SP, Brazil; hector.benatti@gmail.com (H.R.B.); hermesluz@globomail.com (H.R.L.); daniel.magalhaes.lima@gmail.com (D.M.L.); v.dayoub@yahoo.com.br (V.D.G.); franc.borgesma@gmail.com (F.B.C.); vanvanecologia@gmail.com (V.N.R.); 2 Programa de Pós-Graduação em Biotecnologia do Renorbio, Ponto Focal Maranhão, Universidade Federal do Maranhão, São Luís 65080-805, MA, Brazil; 3 Faculdade de Medicina Veterinária, Universidade Estadual do Maranhão, São Luís 65055-970, MA, Brazil; 4 Faculdade de Medicina Veterinária, Universidade Federal de Uberlândia, Uberlândia 38400-902, MG, Brazil; matias.szabo@gmail.com; 5 Faculdade de Medicina Veterinária, Universidade Federal de Mato Grosso, Cuiabá 78060-900, MT, Brazil; danmoura@ufmt.br (D.M.A.); pachecorc@gmail.com (R.C.P.); 6 Embrapa Tabuleiros Costeiros, Aracaju 49025-040, SE, Brazil; ubiratan.piovezan@embrapa.br; 7 Escola Superior de Agricultura Luiz de Queiroz, Universidade de São Paulo, Piracicaba 13418-900, SP, Brazil; katia.ferraz@usp.br

**Keywords:** capybara, human-modified landscapes, natural landscapes, morphometric pattern, biochemical profile

## Abstract

The capybara, *Hydrochoerus hydrochaeris,* is the largest extant rodent of the world. To better understand the correlation between size and body mass, and biochemical parameters of capybaras from areas with different degrees of anthropization (i.e., different food supplies), we sampled free-ranging capybaras from areas of natural landscapes (NLs) and human-modified landscapes (HMLs) in Brazil. Analyses of biometrical and biochemical parameters of capybaras showed that animals from HMLs were heavier (higher body mass) than those from NL, a condition possibly related to fat deposit rather than body length, as indicated by Body Condition Index (BCI) analyses. Biochemical parameters indicated higher serum levels of albumin, creatine kinase, cholesterol, fructosamine and total protein among capybaras from HMLs than from NLs; however, when all adult capybaras were analyzed together only cholesterol and triglycerides were positively correlated with body mass. We propose that the biochemical profile differences between HMLs and NLs are related to the obesity condition of capybaras among HMLs. Considering that heavier animals might live longer and reproduce more often, our results could have important implications in the population dynamics of capybaras among HMLs, where this rodent species is frequently represented by overgrowth populations that generate several levels of conflicts with human beings.

## 1. Introduction

The capybara, *Hydrochoerus hydrochaeris,* is a mammal belonging to the order Rodentia, family Caviidae. It is native to South America, where it occurs in all countries except for Chile [1]. In Brazil, capybaras are widely distributed all over the country, except for its very scarce presence in the semiarid biome, the Caatinga [2,3]. Capybaras are robust, semi-aquatic animals, considered the largest extant rodent in the world, with adults reaching on average between 50 kg and 60 kg and at most 1.35 m in length [3].

Capybaras live in social groups always with more females than males because when male capybaras reach sexual maturity they are expelled from the group by the dominant adult male [3]. The groups live close to water collections—rivers, lakes, flooded regions—because their sweat glands are not well developed [4]; therefore, they need water bodies to regulate body temperature (homeothermy). In addition to thermal regulation, water bodies are a vital resource for capybara, not only for hydration, but also to escape from predators, for mating and for consumption of aquatic plant species [5].

In Brazil, the destruction of native forests for the implantation of monocultures and cultivated pastures has been a historical process over the centuries, especially during the last 50 years [6]. In southeastern Brazil, the expansion of agriculture fields has caused severe defaunation by destroying natural habitats [7]. On the other hand, a few native species have benefited from anthropic alterations, expanding their populations over the human-modified landscapes. One of this species is the capybara [8,9].

The advance of sugarcane crops caused a decrease in the habitat areas of native fauna. Along with these conditions, the state of São Paulo has been experiencing both horizontal and vertical expansion of capybaras populations during the last decades. In the first case, capybaras have started to occupy many areas where they did not exist before; in the second case, population densities have increased significantly in places with already established populations [8,9].

In natural landscapes, as in the Brazilian Pantanal, during the water season capybara select food and ingest smaller quantities, while in the dry season the selection is fewer, but the intake is higher [10]. In the state of São Paulo, where capybaras are close to monocultures such as sugarcane, this behavior of food selection can be reduced, since the amount of energy from the pasture is the main factor when capybaras choose what to eat [11], and the supply of sugarcane is constant throughout the year [12].

Several lines of study have demonstrated that loss or reduction of biodiversity tends to increase the transmission of pathogens and the emergence or reemergence of diseases. This pattern has occurred in several ecosystems, with different pathogens, hosts and vectors. Different studies have confirmed the theory of the correlation between environmental changes and the reduction of biodiversity and disease transmission: West Nile fever [13,14,15], hantavirus [16,17] and Lyme disease [18,19,20], among others. For Brazilian spotted fever (a tick-borne zoonosis transmitted by the capybara tick), Polo et al. [21] demonstrated that the increase and expansion of the sugarcane monoculture was directly related to the territorial expansion of human cases of the disease.

Given the primary importance of capybara in the epidemiology of Brazilian spotted fever and other types of human conflicts, this rodent species is becoming an important object of study, which leads us to believe that a better understanding of its biometric and biochemical profile, and the correlation between both, can be important to better understand the ecological aspects of this rodent among different landscapes. To better understand the correlation between size and body mass, and biochemical parameters of capybaras from areas with different degrees of anthropization (i.e., different food supplies), in the present study we sampled free-ranging capybaras from areas of natural landscapes (NLs) and human-modified landscapes (HMLs). Our main hypothesis is that the high food supply among HMLs would impact on the body conditions and healthy status of capybaras when compared to the NLs in which capybaras live under minimal anthropic interference.

## 2. Materials and Methods

### 2.1. Study Areas

Captures of free-ranging capybaras were carried out during 2015–2019 in seven municipalities of the state of São Paulo (HMLs), and in two municipalities (NLs), one in the state of Mato Grosso and another one in the state of Mato Grosso do Sul (Table 1). Among HMLs, sampled capybaras belonged to groups that were established adjacent to or within human settlements, such as farms, university campuses and public or private parks, where capybaras had constant access to artificial pastures and crops. In NLs, sampled capybaras belonged to groups established in areas with no artificial pastures or crops, and with minimal anthropic changes during the last few decades.

### 2.2. Animal Capture and Containment

Capybaras were captured by using 16 to 20 m^2^ corrals baited with sugar cane and green corn. Once closed in the corral, animals were physically restrained by a net catcher and anaesthetized with an intramuscular injection of a combination of ketamine (10 mg/kg) and xylazine (0.2 mg/kg). In Mato Grosso do Sul, corrals were not effective; thus, capybaras were captured by anesthetic darting via a CO₂-injection rifle (Dan-Inject model JM Standard, Denmark) using the same chemicals listed above [22]. Under anesthesia, animals were subjected to biometrical measures (described below) and identified with a subcutaneous microchip (Alflex model P/N 860005–001, Capalaba, Australia). After recovering from anesthesia, capybaras were released at the same capture site, as described elsewhere [22].

### 2.3. Biometrical Measures

Only adult animals weighing > 35.0 kg [3] were measured. Young animals were not sampled because, at each stage of growth and development, the proportional or absolute amount of energy reserves can be expected to change with normal growth processes, even in an ideal environment [23]. For each sampled capybara, we measured the length, height and perimeter (neck, thorax and abdomen) of the body (in centimeters), as well as the body mass (kg), as commonly applied to biometric studies of rodents [24,25].

**Figure 1:**
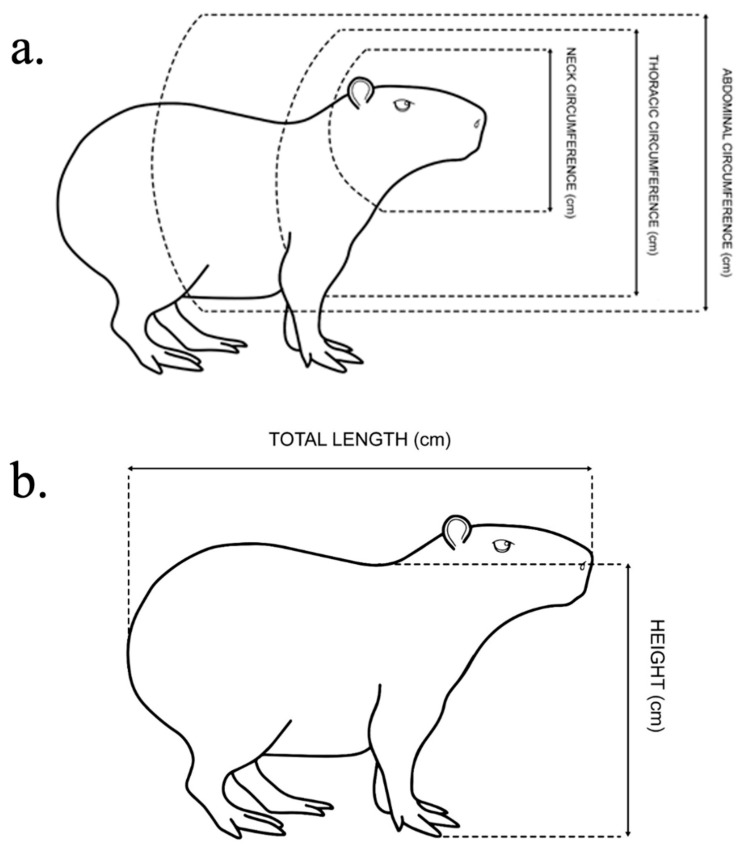
Representation of the measures taken from the capybaras. Linear measurements (**a**), and circumference measurements (**b**).

The total length of the animals was measured from the end of the muzzle to the end of the last vertebra, and the height was measured from the highest point of the withers (with the capybara’s paw stretched, measured from the tip of the largest finger to the upper margin of the scapula). The neck perimeter was measured at the angle of the mandible. The chest perimeter was measured at the height of the armpit and the circumference of the abdomen at the umbilical line (Figure 1). For all measures polyester tape 150 cm long and with 1 cm precision was used (Corrente Coats, Brazil).

Weighing was done with the animal wrapped in a nylon net (weight 2.4 kg). The scale used was a digital dynamometer with a precision of 0.1 kg (Pesola model PCS0300, Hatton Rock, UK); it was attached to the net through a metal hook and then the net/animal set was suspended using a 1.5 m-aluminum bar.

### 2.4. Definition of Body Condition Index (BCI)

It has been pointed out that the calculation of the body condition of animals is a common goal for several studies in order to compare absolute size (or other measurement) with body mass; however, authors have used several terminologies to indicate the same thing [25,26]. In the present work, the adopted terminology was the “body condition index” (BCI), which was firstly proposed by Hepp et al. (1986) [27]. To our knowledge, information on body condition index (BCI) for the evaluation of large rodents is absent in the literature. Therefore, five ratios previously applied to small rodents and other mammals were extrapolated for capybaras, as follows:

- BM/TL: Body mass (BM) divided by total length (TL) [28,29].
- BM/TL^2^: Body mass (BM) divided by the square of the total length (TL^2^) [30,31].
- BM/TL^3^: Body mass (BM) divided by the cube of the total length (TL^3^) [29].
- resBM/TL^2^: Residual of linear regression of body mass (resBM) divided by the square of total length (TL^2^) [32].
- resBM/TL^3^: Residual of the linear regression of the body mass (resBM) divided by the cube of the total length (TL^3^) [33,34].

### 2.5. Fat Amount Estimative

An assumption of fat amount was estimated by calculating the residual index, which is the regressed body mass over body size. The residual index was calculated after data were appropriately transformed to meet regression assumptions. The residual distance from the point of the individual to the regression curve works as an estimate of the body condition, i.e., the distance from the point to the curve can be a good estimate of the extra mass accumulation, in this case, the fat [35].

### 2.6. Blood Collection and Serum Biochemistry

Blood samples (5 to 10 mL of venous blood) were collected through the cranial cava, saphenous or cephalic vein of each adult capybara. These samples were immediately stored in a vacuum tube containing separator gel for subsequent serum centrifugation. In the laboratory, sera were transferred to two 1.5 mL polypropylene tubes in order to keep all the samples in duplicates, and then frozen at −20 °C for further analyses.

Biochemical variables were determined on a Labtest^®^ biochemical analyzer, Labmax 240 model. To verify the presence of possible obesity-related metabolic disorders, the biochemical profile of adult capybaras was performed. The following serum elements were analyzed: Triglycerides, cholesterol, fructosamine, albumin, total protein, aspartate aminotransferase, alkaline phosphatase, creatine kinase, urea, calcium and phosphorous. All protocols were performed according to the manufacturer’s instructions of each kit.

For liver profile, measurements of aspartate aminotransferase (BioSystems^®^–11531, Barcelona, Spain) and alkaline phosphatase (BioSystems^®^–11593, Barcelona, Spain) were performed. Obese patients tend to develop liver steatosis, which would lead to increases in liver enzymes, aspartate aminotransferase (AST) and alkaline phosphatase (ALP). Even if there is no clinical condition of steatosis, the simple deposition of excess fat can lead to increases in these enzymes [36].

For renal profile, measurements of urea (BioSystems^®^–11541, Barcelona, Spain) were performed. Obese patients tend not to have large changes in serum urea concentrations, but since the intent is an overview of the animal’s general picture, it is important to determine the animal’s renal function. Urea is directly related to muscle mass and obese patients have less muscle mass, which may lead to decreased or fluctuating serum urea levels [36].

To analyze fat metabolism, measurements of cholesterol (Labtest^®^–76-2/100, Minas Gerais, Brazil) and triglycerides (Labtest–87-2/250, Minas Gerais, Brazil) were performed. These parameters were chosen because they tend to be increased in obese animals [36]. When increased, they generate insulin resistance, or a diminished cellular response to a given plasma insulin concentration [37]. Insulin, one of the main anabolic hormones, has diminished action when triglycerides and cholesterol are increased, resulting in increased muscle catabolism rates, and consequently leading to increased serum creatine kinase (CK) [38]. Thus, CK levels were also analyzed (ByoSystems^®^–21790, Barcelona, Spain).

Because of the possible insulin resistance in obese animals, we also measured the amount of serum fructosamine (ByoSystems^®^–11046, Barcelona, Spain). Fructosamine is glucose conjugated with blood albumin [39]. Fructosamine gives a seven- to ten-day overview of blood glucose behavior. Since obese patients have insulin resistance, there is a possibility of increased fructosamine [40].

Protein metabolism analyses were performed by measuring total protein (Labtest^®^—99-250, Minas Gerais, Brazil) and albumin (Labtest^®^—19-1/250, Minas Gerais, Brazil). Albumin is related to fructosamine. In addition, the World Health Organization considers obesity to be a pro-inflammatory or inflammatory state [41], and albumin is an acute, but a negative, phase inflammatory protein, that is, in an inflammation picture given by obesity, albumin falls, recording a serum decrease in values, as well as a possible fluctuation in total protein measurement [36].

Overweight patients have metabolic dysfunction, which may lead to alterations in the hypothalamus-pituitary-adrenal axis, leading to increases in cortisol. Cortisol, by definition, is a catabolic hormone, causing increased muscle catabolism rates, which, again, may increase serum CK concentrations. Increased blood CK may cause proximal tubular damage to the kidneys, causing changes in serum phosphorus levels (Labtest^®^–12-200, Minas Gerais, Brazil) [38].

Finally, obese animals have lower bone and muscle densities when compared to non-obese individuals, so there might be changes in the total blood calcium concentrations of obese animals, registering lower levels [42]. Because of this, a serum calcium measurement was performed (BioSystems^®^—11570, Barcelona, Spain).

### 2.7. Statistical Analyses

Biometrical and biochemical data were evaluated using descriptive statistics, using violin-type density graphs and statistical summaries describing the groups through means and standard deviations for group comparison (HMLs versus NLs). Differences between capybaras from HMLs and NLs were assessed using the Student’s *t*-test for parametric distributions or non-parametric Wilcoxon test. Normality was verified by the Shapiro–Wilk test and for all analyses 5% was used as the significance level. For statistical analysis, by standardization, only the most recent recaptures were included in the calculations. Preliminarily, data were analyzed considering all adult capybaras (males + females) or only adult female capybaras (the majority of the sampled animals). As variables presenting statistically significant differences were the same by both analyses, only the data with all adult capybaras is presented.

Correlation between serum biochemical data (triglycerides, cholesterol, fructosamine, albumin, total protein, aspartate aminotransferase, alkaline phosphatase, creatine kinase, urea, calcium and phosphorous) and capybara body mass or BCI values were tested by linear regression analyses, considering each biochemical variable independently. For this purpose, adult capybaras were analyzed all together, regardless of whether they were from HMLs or NLs.

All analyses were performed using R software (R Core Team, 2017, Vienna, Austria).

## 3. Results

### 3.1. Capybara Samples

A total of 218 adult capybaras were sampled, 174 in HMLs and 44 in NLs (Table 2). Among these, 52 were recaptures. For statistical comparison, a total of 134 capybaras were analyzed in HMLs and 32 in NLs; total: 166 adults (39 males, 127 females).

### 3.2. Biometrics and Body Condition Index (BCI)

Overall, the body mass range of adult capybaras ranged from 35 kg to 105.6 kg. Among HMLs, the mean body mass was 54.5 kg (range: 35.4 kg–83.4 kg) for males, and 63.1 kg (35 kg–105.6 kg) for females. Notably, three female capybaras weighed 105.6, 104.8, and 100.6 kg, and were the overall heaviest animals, all captured in the HML of Pirassununga municipality. Among NLs, the mean body mass was 48.6 kg (range: 38.6 kg–63.6 kg) for males and 54.8 kg (35.7 kg–80.4 kg) for females. Overall, mean weights for adult capybaras were 61.2 kg for HMLs and 54.2 kg for NLs.

**Figure 2:**
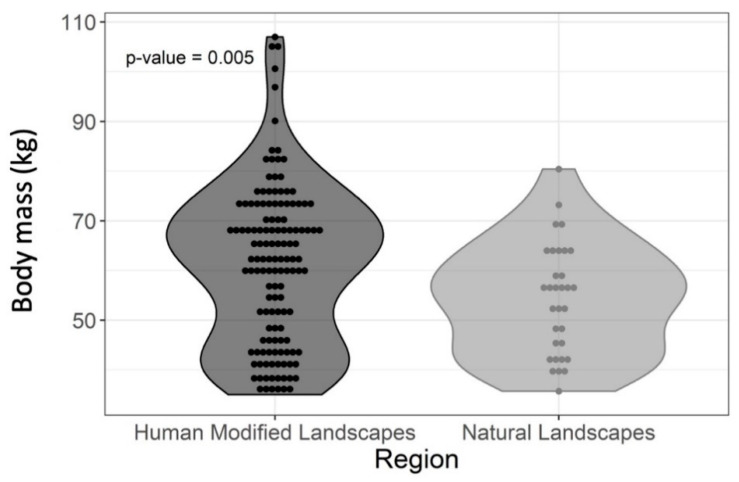
Graphical representation of individual body mass values of adult capybaras according to the study region (landscape type).

**Figure 3:**
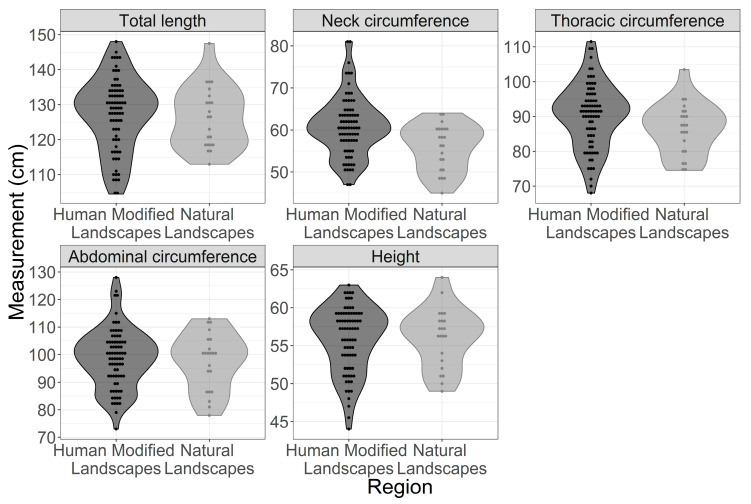
Graphical representation of individual values of five types of body measurement of adult capybaras, according to the study region (landscape type).

Within the same experimental group (HML or NL), there were no significant differences between body mass and body measures of male and female capybaras. The body mass of capybaras from HMLs was, on average, 7 kg higher than the body mass of animals from NLs (*p* = 0.005). In contrast, there were no significant differences (*p* > 0.05) in body height and total length to explain the body mass differences (Figure 2 and Figure 3; Table 3).

**Figure 4:**
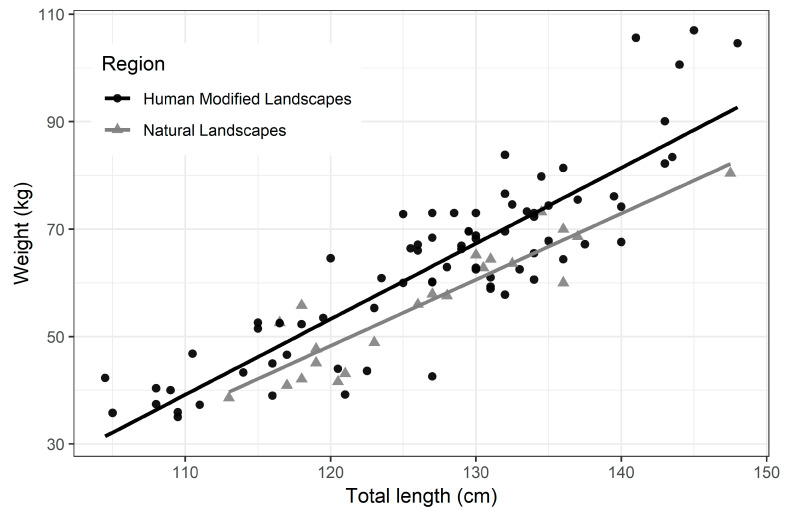
Graphical representation of the distribution of body mass in relation to the total body length of adult capybaras from human-modified landscapes and natural landscapes in Brazil. Data were significantly different (linear regression; *p* = 0.001) between HML and NL.

By performing body mass regression as a function of total body length, height and gender, only the total length explained a significant portion of body mass variance. Thus, the residue of body mass regression as a function of total length functions as an approximation of the amount of fat deposited, an important variable in body mass composition (Figure 4). Note: The r^2^ of both models was about the same, 75%. Regarding the circumference measurements between capybaras from HMLs and NLs, statistical differences were observed for neck and chest, but not for abdominal circumference (Table 3).

**Figure 5:**
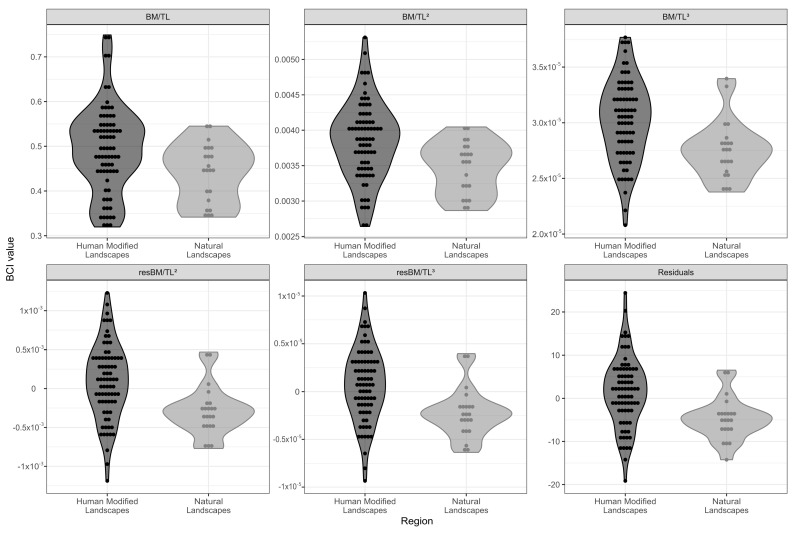
Graphical representation of individual values of five types of Body Condition Index (BCI) for adult capybaras, according to the study region (human-modified landscapes and natural landscapes).

By comparing all the BCI, animals from HMLs had significantly higher BCI than animals from NLs (Table 4, Figure 5). Regardless of the formula used to calculate the BCI, there were always differences between the two groups, with higher body index for animals from HMLs.

### 3.3. Blood Biochemistry

The biochemical variables were measured in a total of 161 free-ranging adult capybaras, 129 from HMLs and 32 from NLs. The results of the statistical analyses of the biochemical parameters compared between the two groups (HMLs and NLs) are shown in Table 5. Biochemical parameters such as ALP, phosphorus, triglycerides and urea showed no statistical differences (*p* > 0.05) between the two capybara groups. Significant differences (*p* < 0.05) between animals from HMLs and NLs were observed for albumin, CK, cholesterol, fructosamine and total protein. Some parameters were borderline: Calcium (*p* = 0.0523) and AST (*p* = 0.0597).

### 3.4. Regression Analysis between Capybara Body Mass or BCI and Biochemical Parameters

By analyzing all capybaras from HMLs and NLs together, we evaluated possible correlations between body mass with biochemical parameters through linear regression. In this regard, there was no significant relationship (*p* > 0.05) between body mass and the parameters fructosamine, total protein, AST, CK, urea, calcium or phosphorous. However, there were positive correlations (*p* < 0.05) between capybara body mass and factors related to fat metabolism, namely, cholesterol and triglycerides. In addition, there was a negative correlation (*p* < 0.05) between body mass and the amount of circulating albumin or ALP (Table 6).

Similarly to body mass, some of the BCI types were also positively correlated (*p* < 0.05) with cholesterol (BM/TL^2^) and triglycerides (BM/TL and BM/TL^2^). In addition, there were negative correlations (*p* < 0.05) between the amount of circulating phosphorous and all five BCI types, and between AST and all BCI types but BM/TL^3^ (Supplementary Tables S1–S5). 

## 4. Discussion

Analyses of biometrical and biochemical parameters of capybaras from HMLs and NLs pointed out to significant differences between these two groups, with animals from HML being heavier (higher body mass) than those from NL, although the body total length and height did not differ statistically. Biochemical parameters indicated significant differences between animals from HMLs and NLs for albumin, CK, cholesterol, fructosamine and total protein; however, when all adult capybaras were analyzed together only cholesterol and triglycerides were positively correlated with body mass, and albumin negatively correlated with body mass. Our samples of adult capybaras were characterized by a predominance of females over males, in either HMLs or NLs. This condition is in agreement with the typical social structure of capybara groups [3]. Anyhow, our analyses considered females and males together because our preliminary analyses did not show any significant differences between males and females within the same experimental group (HML or NL).

Previous records of body mass of adult capybaras from areas with low anthropic alterations, such as the Venezuelan Llanos, reported a 35 kg–65 kg range [43], whereas one study from the Brazilian Pantanal reported adult capybaras weighting up to 70 Kg [1]. Our records from NLs (range: 35 kg–79.8 kg; mean: 54.2 kg) are similar to these previous reports. In contrast, our records from HMLs in the state of São Paulo (range 35 kg–105.2 kg; mean: 61.2 kg) are much higher, and at the same time, similar to a previous report for adult female capybaras in an HML in the state of São Paulo: Range 48.0 kg–81.0 kg; mean 62.0 kg [44]. Noteworthy, we report three capybaras weighing more than 100 Kg, which to our knowledge comprise the highest values for the species. Previously, Moreira et al. (2013) [3] reported a maximum body mass value of 100 kg, with no indication of geographic origin.

The five body indexes showed differences between the sampled groups, revealing that capybaras in the HMLs had higher BCI than capybaras in the NLs and therefore higher fat reserves. We can infer that the extra weight of animals from HMLs comes from excess fat, since most of the fat in a capybara is subcutaneous [45]. The measurements of the perimeters indicated higher fat deposits in animals in the HMLs. Even though Jakob et al. [26] stated that results vary dramatically according to the BCI chosen to calculate and to analyze the body condition, in the present study all five BCIs showed significant differences between HMLs and NLs. Since body condition is treated as a measure of energy status, animals in better conditions for survival are expected to have higher energy reserves (usually fat) [46].

Based on individual energy reserves, we can infer that capybaras from HMLs have better survival conditions than animals from NLs. Firstly, this is due to the vast supply of food offered by crops (e.g., sugarcane, corn) and cultivated grasses throughout the state of São Paulo [9]. Secondly, besides the presence of pumas (*Puma concolor*), a natural predator of capybaras in HMLs [47], heavier animals are able to some extent to avoid predation, despite having impaired locomotion [48]. In addition to the possible better survival conditions of capybaras in HMLs, it is known that both natural and sexual selection tend to favor larger and heavier individuals [49,50]. The obvious, although fundamental, generalization is that the greater the availability of limiting resources, the larger the population size [3]. A trait that responds to food availability, such as body mass, is likely to covary with fitness at the phenotypic level; i.e., individuals that have access to more food recourses are heavier and reproduce more [51].

There are studies with domestic cattle considering body condition and reproductive efficiency, the higher the BCI, the better the reproductive efficiency [52]. This association stems from the fact that the corpus luteum might be directly activated by cholesterol, a progesterone precursor [53]. There are also reports of a relationship between blood fatty acids and prostaglandin production. The direct effect of positive energy balance (energy consumed minus energy required for maintenance) on luteinizing hormone secretion has been demonstrated in heifers for over 30 years [54]. Therefore, there is a great possibility that capybaras in HMLs, due to access to abundant food supply, are reproducing on larger scales than the animals of NLs, thus forming larger groups.

Regarding the blood biochemistry patterns, the present ALP values (HMLs = 151.1 ± 104.7 U/L; NLs = 171.5 ± 163.9 U/L) were lower than the values reported for free ranging capybaras in Paz de Ariporo (235.75 ± 144.83 U/L), Colombia [55]. The reasons for overall lower ALP values in the present study are unknown, and could be related to the unexpected negative correlation between body mass and ALP, when all capybaras were analyzed together. The values of phosphorus (HMLs = NLs = 5.5 ± 2 mg/dL) and triglycerides (HMLs = 83.6 ± 100.4 U/L; NLs = 76 ± 72.7 U/L) are similar to those reported by Matus and Pulido [56] for captive capybaras (5.081 ± 3.99 mg/dL for phosphorus and 89.15 ± 89.24 U/L for triglycerides). 

Endocrinological studies indicate that overweight and obese populations have lower serum concentrations of vitamin D, due to its sequestration by adipose tissue [57]. Vitamin D has, as one of its functions, increasing serum calcium and phosphorus levels [58]. Interestingly, while there was no statistical association between any BCI type and calcium, there were negative correlations for all five types of BCI and phosphorus levels when all capybaras in the present study were analyzed together. Such negative associations are likely related to low intake of this mineral since, if it were physiological, we would also observe a decrease in calcium levels. 

The urea was compared with results available in the literature by converting the results found by our group to blood urea nitrogen (BUN), which consists of multiplying the value found for serum urea by 0.467 (stoichiometric calculation; molecular weights: Urea 60.06 g/mol; nitrogen 14 g/mol), since that found in the literature for the species is the BUN. Therefore, converted from urea to BUN we have the following results: HMLs = 12.18 ± 7.79 mg/dL; NLs = 13.3 ± 4.85, which in both cases, are above the results recorded by Matus and Pulido [56].

The values found for serum albumin in this study (HMLs = 2.8 ± 0.4 g/dL; NLs = 2.5 ± 0.4 g/dL) are close to those found in free-living capybaras (2.54 ± 0.25 g/dL) by Álvarez-Méndez and Barragán [55]; however, they differ from the values recorded by Matus and Pulido [56] for captive capybaras (3.175 ± 0.28 g/dL). Normally, the increase in serum albumin can be directly attributed to the contribution in the diet [36]. Since HML-capybaras are exposed to an unrestricted food supply [59], this could justify the significantly higher albumin levels recorded in these animals when compared to NLs. On the other hand, when all adult capybaras were analyzed together, there was a negative correlation between body mass and serum albumin. This result is explained by the fact that albumin is an acute, but a negative, phase inflammatory protein that tends to have decreased levels at obesity conditions [36].

Notwithstanding the difference in diet, capybaras from NLs are subjected to the presence of natural predators—jaguar (*Panthera onca*), puma (*Puma concolor*), caiman alligators (*Caiman* spp.) and anacondas (*Eunectes* spp.) [3,60,61,62]—while animals in HMLs have only the puma as a natural predator, although at lower magnitude than among NLs [47]. The presence of predators exerts an ‘intimidation’ effect on potential prey, which in the case of the present study, is the capybara. This intimidation effect may lead to changes in the hypothalamic–pituitary–adrenal axis, generating a picture of chronic stress [63]. This intimidation impairs the foraging of herds, preventing animals from taking full advantage of available food [64]. In addition to the primary stress caused by fear of predation, another determining factor for the animal’s physical condition is directly related to the fact described above about feeding efficiency. Physiological changes may be observed in association with variation in food intake [65], food demand [66] and conversion efficiency [67], all of which may be affected by the presence of predators.

Previous studies dealing with stress-associated changes in plasma immunoglobulin levels demonstrated that chronic stress increases blood levels of immunoglobulins, leading to an increase in total protein [68,69]. In addition, the capture myopathy can generate significant increases in blood CK levels [70]. In the study conducted by Álvarez-Méndez and Barragán [55], capybaras were restrained for a few minutes without anesthesia, only for physical examination and blood collection. In the case of our study, capybaras from HMLs were captured in corrals, sometimes at large groups with >20 individuals per capture. Therefore, some individuals remained restrained in this condition for hours until being sampled. This may justify the increased CK levels of capybaras of the HMLs, due possibly to stress and capture myopathy, when compared to NLs where capybaras were captured by darting or at smaller groups in corrals.

In our study, NL capybaras had a serum cholesterol value (37.6 ± 15.4 mg/dL) close to those recorded by Matus and Pulido [56] for captive capybaras (39.78 ± 26.67 mg/dL). However, capybaras sampled in HMLs showed serum cholesterol values quite higher (50.7 ± 16.7 mg/dL). This is possibly related to the calorie-restricted diet that the capybaras from NLs and those from the study by Matus and Pulido [56] were subjected to. In NLs of the present study, capybaras did not have access to a high-calorie diet such as crops [59,71], while HML capybaras had free access to several crop fields, such as sugar cane and corn, highly energetic sources of food [59,71]. Interestingly, a laboratory study demonstrated that Fischer 344 rats exposed to non-restricted diets had higher serum cholesterol levels than rats exposed to dietary restrictions [40]. These results are corroborated by the positive correlations of body mass and cholesterol levels, and of BCI and cholesterol levels, when all capybaras of the present study were analyzed together. Similar interpretation can be given to the level of triglycerides, which is also known to be increased in obese animals [36], and was also positively correlated with body mass and some BCI types in the present study.

To our knowledge, this is the first work to measure fructosamine in capybaras. As previously mentioned, fructosamine gives a seven- to ten-day overview of blood glucose behavior. Since obese patients have insulin resistance, our hypothesis that capybaras from HMLs, exposed to high-calorie diets, have higher fructosamine values than capybaras from NLs [40], is confirmed in the present study.

As previously defined, aspartate aminotransferase (AST) increases due to cell death for several reasons, including hepatic steatosis, but it can also increase due to muscle damage [36]. A recent study [59] demonstrated that daily movements of capybaras were lower among HMLs than in NLs. This decrease in the patterns of displacement and activity can generate long periods in which the animals remain in decubitus in the HMLs, justifying the borderline increase in AST due to muscle injury. In addition, higher AST values in the HMLs could also be related to the presence of infection by the liver fluke, *Fasciola hepatica,* in capybaras of at least two of the HMLs of the present study (Piracicaba and Tatuí), as recently reported [72]. This could also be the reason for the negative correlations between AST and most of the BCI types, considering that the nine highest values of AST of the present study were from animals from Piracicaba and Tatuí, with mean body mass of 52 Kg (data not shown), below the overall mean body mass of 61.2 Kg for the HMLs. In fact, if we remove just the single highest AST value from our analyses, there would be no significant association between AST and any of the BCI types (data not shown). 

## 5. Conclusions

This study demonstrated that there were significant differences for some biometrical and biochemical parameters of capybaras between HMLs and NLs. In HMLs, where more energy-rich food sources are available for capybaras, their higher body mass is related to fat deposit rather than body length, as indicated by BCI analyses. The presumed obesity condition among HMLs could also be the reason for higher values of some biochemical parameters that are usually related to obesity, such as higher blood levels of cholesterol, triglycerides and fructosamine, when compared to capybaras from NLs. Considering that heavier animals could live longer and reproduce more often, the present results could have important implications in the population dynamics of capybaras among HMLs, where this rodent species is frequently represented by overgrowth populations that generate several levels of conflicts with human beings [8,73,74,75,76]. In this sense, the re-emergence of Brazilian spotted fever—a highly fatal tick-borne bacterial disease—among HMLs in the state of São Paulo has been linked to increased capybara population and reproduction rates since capybaras are major hosts of the tick vector, *Amblyomma sculptum* [22,76,77,78].

## Figures and Tables

**Table 1:** Location of capybara captures and sampling.

Location	Municipality/State	Approximate Coordinates
Human Modified Landscapes (HMLs)		
University of São Paulo campus of Piracicaba	Piracicaba/SP	22°43′05.1″ S 47°36′39.6″ W
Carioba sewage treatment station	Americana/SP	22°42′35.7″ S 47°20′24.5″ W
Federal University of São Carlos campus of Araras	Araras/SP	22°18′34.4″ S 47°23′00.2″ W
Private Company	Tatuí/SP	23°22′42.4″ S 47°55′01.0″ W
University of São Paulo campus of Pirassununga	Pirassununga/SP	21°56′52.4″ S 47°27′13.6″ W
University of São Paulo campus of Ribeirão Preto	Ribeirão Preto/SP	21°10′03.9″ S 47°51′30.7″ W
Avaré State Park	Avaré/SP	23°05′59.2″ S 48°54′32.9″ W
Natural Landscapes (NLs)		
Pantanal of Poconé	Poconé/MT	16°29′35.6″ S 56°26′20.8″ W
Pantanal of Nhecolândia	Corumbá/MS	19°14′53.5″ S 57°01′34.0″ W

**Table 2:** Number of adult capybaras sampled by locality of human-modified landscapes (HMLs) and natural landscapes (NLs) in Brazil.

Groups	Municipality	Number of Adult Capybaras				
		Total Sampled	Recaptures	Included in the Study		
				Males	Females	Total
HMLs	Piracicaba	38	13	3	22	25
	Americana	12	2	5	5	10
	Araras	17	2	1	14	15
	Tatuí	14	0	3	11	14
	Pirassununga	66	13	14	39	53
	Ribeirão Preto	22	10	4	8	12
	Avaré	5	0	1	4	5
	TOTAL	174	40	31	103	134
NLs	Poconé	20	10	4	6	10
	Corumbá	24	2	4	18	22
	TOTAL	44	12	8	24	32

HMLs—human-modified landscapes; NLs—natural landscapes.

**Table 3:** Comparison of biometric parameters between capybaras from human-modified landscapes (HMLs) and natural landscapes (NLs).

Measurement	Region	Mean (SD)	Median (IQR)	*p*-Value
Height	HMLs	55.9 (4.3)	57 (5.9)	0.8
	NLs	56.1 (3.9)	56.8 (5.1)	
Total length	HMLs	127.3 (10.3)	129.2 (13.6)	0.69
	NLs	126.4 (8.7)	126.5 (13.1)	
Abdominal circumference	HMLs	98.7 (10.7)	99.5 (11.8)	0.6578
	NLs	97.6 (10.3)	100.2 (15.5)	
Neck circumference	HMLs	60.9 (7.1)	60.8 (6.8)	0.000966
	NLs	55.9 (5.5)	57.2 (8.5)	
Thoracic circumference	HMLs	90.4 (9.3)	91.5 (11.6)	0.049
	NLs	86.5 (7.4)	87.5 (10.1)	
Body mass	HMLs	61.2 (16.1)	62.7 (25.8)	0.005
	NLs	54.2 (11.2)	55.7 (18.4)	

SD: Standard deviation; IQR: Interquartile range.

**Table 4:** Body Condition Index average values of capybaras from human-modified landscapes (HMLs) and natural landscapes (NLs).

Formula	Region	Mean (SD)	*p*-Value
BM/TL	HMLs	0.4928 (0.0965)	0.005
	NLs	0.4410 (0.0656)	
BM/TL^2^	HMLs	0.0038 (0.0005)	<0.001
	NLs	0.0035 (0.0004)	
BM/TL^3^	HMLs	3.03 × 10^5^ (3.64 × 10^3^)	<0.001
	NLs	2.75 × 10^5^ (2.62 × 10^3^)	
Residue	HMLs	1.37 (8.01)	<0.001
	NLs	−4.74 (4.89)	
resBM/TL^2^	HMLs	8.75 × 10^5^ (0.00048)	<0.001
	NLs	−2.9 × 10^11^ (0.00031)	
resBM/TL^3^	HMLs	71.8 (3.79 × 10^3^)	<0.001
	NLs	−2.28 × 10^3^ (2.57 × 10^3^)	

BM/TL: Body mass (BM) divided by total length (TL); BM/TL^2^: Body mass (BM) divided by the square of the total length (TL^2^); BM/TL^3^: Body mass (BM) divided by the cube of the total length (TL^3^); resBM/TL^2^: Residual of linear regression of body mass (resBM) divided by the square of total length (TL^2^); resBM/TL^3^: Residual of the linear regression of the body mass (resBM) divided by the cube of the total length (TL^3^).

**Table 5:** Comparisons of biochemical parameters between capybaras from human-modified landscapes (HMLs) and natural landscapes (NLs).

Serum Biochemical Parameter	Region	Mean (SD)	Median (IQR)	*p*-Value
Albumin (g/dL)	HMLs	2.8 (0.4)	2.9 (0.6)	6.00 × 10^−4^
	NLs	2.5 (0.4)	2.5 (0.7)	
Alkaline Phosphatase (U/L)	HMLs	151.1 (104.7)	115.3 (148.2)	0.7756
	NLs	171.5 (163.9)	92.8 (244.9)	
Aspartate Aminotransferase (U/L)	HMLs	21.7 (25)	14 (8.4)	0.0597
	NLs	13.6 (6.9)	11.8 (10.4)	
Calcium (mg/dL)	HMLs	10.3 (1.2)	10.5 (1.3)	0.0523
	NLs	10.9 (0.9)	10.8 (1)	
Creatine Kinase (U/L)	HMLs	261 (733.2)	87.3 (100.3)	<0.0001
	NLs	61.4 (77)	35 (48.2)	
Cholesterol (mg/dL)	HMLs	50.7 (16.7)	47.1 (14.5)	<0.0001
	NLs	37.6 (15.4)	34.1 (16.2)	
Fructosamine (mmol/L)	HMLs	200.9 (53.5)	190 (68)	0.0332
	NLs	175.6 (33.9)	175 (42)	
Phosphorous (mg/dL)	HMLs	5.5 (2)	5 (2.5)	0.9901
	NLs	5.5 (2)	5.4 (2.3)	
Total Protein (g/dL)	HMLs	6 (0.6)	6 (0.7)	0.0137
	NLs	6.3 (0.6)	6.5 (1)	
Triglycerides (mg/dL)	HMLs	83.6 (100.4)	50.9 (57.4)	0.9345
	NLs	76 (72.7)	55.9 (61)	
Urea (mg/dL)	HMLs	26.1 (16.7)	24.2 (19.4)	0.2498
	NLs	28.6 (10.4)	25.4 (10.5)	

SD: Standard deviation; IQR: Interquartile range.

**Table 6:** Results of regression analysis between body mass and biochemical parameters among adult capybaras (animals from human-modified and natural landscapes were all analyzed together).

Parameters	Regression Equation	R^2^ (%)	*p* Value
Fructosamine (Fru)	Fru (mmol/L) = 190.1 + 0.1701 body mass	0.23	0.601
Total protein (TP)	TP (g/dL) = 4769 − 3.29 body mass (kg)	0.03	0.829
Aspartate aminotransferase (AST)	AST (U/L) = 33.55 − 0.2299 body mass (kg)	2.10	0.083
Alkaline phosphatase (ALP)	ALP (U/L) = 265.1 − 1.863 body mass (kg)	6.07	0.007
Creatine kinase (CK)	CK (U/L) = 2085 − 25.12 body mass (kg)	0.89	0.306
Urea (Ur)	Ur (mg/dL) = 32.38 − 0.07457 body mass (kg)	0.54	0.381
Calcium (Ca)	Ca (mg/dL) = 10.86 − 0.006089 body mass (kg)	0.61	0.351
Phosphorous (P)	P (mg/dL) = 5.871 − 0.01028 body mass (kg)	0.65	0.337
Cholesterol (Cho)	Cho (mg/dL) = 25.64 + 0.3397 body mass (kg)	13.73	<0.001
Triglycerides (Tri)	Tri (mg/dL) = −47.97 + 2.046 body mass (kg)	12.2	<0.001
Albumin (Alb)	Alb (g/dL) = 3.111 − 0.005519 body mass (kg)	4.20	0.014

## Data Availability

The data presented in this study are available on reasonable request from the corresponding author. The data is not publicly available as not all data from the study has been published yet.

## Supplementary Materials

The following are available online at https://www.mdpi.com/article/10.3390/vetsci8080165/s1, Table S1: Results of regression analysis between the Body Condition Index (BCI: BM/TL)* and biochemical parameters among adult capybaras (animals from human-modified and natural landscapes were all analyzed together); Table S2: Results of regression analysis between the Body Condition Index (BCI: BM/TL^2^)* and biochemical parameters among adult capybaras (animals from human-modified and natural landscapes were all analyzed together); Table S3: Results of regression analysis between the Body Condition Index (BCI: BM/TL^3^)* and biochemical parameters among adult capybaras (animals from human-modified and natural landscapes were all analyzed together); Table S4: Results of regression analysis between the Body Condition Index (BCI: resBM/TL^2^)* and biochemical parameters among adult capybaras (animals from human-modified and natural landscapes were all analyzed together); Table S5: Results of regression analysis between the Body Condition Index (BCI: resBM/TL3)* and biochemical parameters among adult capybaras (animals from human-modified and natural landscapes were all analyzed together).
