# Supplementary material for: Morphometric Patterns and Blood Biochemistry of Capybaras (Hydrochoerus hydrochaeris) from Human-Modified Landscapes and Natural Landscapes in Brazil
Source: Vet Sci. 2021 Aug 13;8(8):165. doi: 10.3390/vetsci8080165 (PMC8402786; doi:10.3390/vetsci8080165)
Supplement: Supplementary file 1 [file vetsci-08-00165-s001.zip › vetsci-1298644-supplementary.pdf]

Supplementary Table S1. Results of regression analysis between the Body Condition Index (BCI: BM / TL)* and biochemical parameters among adult capybaras (animals from human-modified and natural landscapes were all analyzed together).

**Parameters**	**Regression equation**	**R^2 (%)**	***P* value**
Fructosamine (Fru)	Fru (mmol/L) = 174.4 + 17.52 BCI	0.07	0.858
Total protein (TP)	TP (g/dL) = 6.151 + 0.102 BCI	0.02	0.930
Aspartate aminotransferase (AST)	AST (U/L) = 97.99 - 144.8 BCI	12.70	0.013
Alkaline phosphatase (ALP)	ALP (U/L) = 304.8 - 279.2 BCI	5.78	0.152
Creatine kinase (CK)	CK (U/L) = 9450 - 16954 BCI	5.34	0.114
Urea (Ur)	Ur (mg/dL) = 47.33 - 30.93 BCI	1.88	0.353
Calcium (Ca)	Ca (mg/dL) = 9.032 + 2.703 BCI	4.10	0.168
Phosphorous (P)	P (mg/dL) = 9.537 - 7.817 BCI	8.22	0.048
Cholesterol (Cho)	Cho (mg/dL) = 23.86 + 50.37 BCI	7.12	0.067
Triglycerides (Tri)	Tri (mg/dL) = - 149.2 + 506.1 BCI	11.18	0.020
Albumin (Alb)	Alb (g/dL) = 3.076 - 0.6885 BCI	1.78	0.367

* BM / TL: Body mass (BM) divided by total length (TL)

Supplementary Table S2. Results of regression analysis between the Body Condition Index (BCI: BM / TL^2)* and biochemical parameters among adult capybaras (animals from human-modified and natural landscapes were all analyzed together).

**Parameters**	**Regression equation**	**R^2 (%)**	***P* value**
Fructosamine (Fru)	Fru (mmol/L) = 186.7 - 1033 BCI	0.01	0.951
Total protein (TP)	TP (g/dL) = 6.481 - 75.1 BCI	0.32	0.702
Aspartate aminotransferase (AST)	AST (U/L) = 122.8 - 25303 BCI	13.28	0.011
Alkaline phosphatase (ALP)	ALP (U/L) = 271.9 - 26916 BCI	1.80	0.428
Creatine kinase (CK)	CK (U/L) = 13595 - 3293030 BCI	6.89	0.071
Urea (Ur)	Ur (mg/dL) = 52.38 - 5335 BCI	1.91	0.349
Calcium (Ca)	Ca (mg/dL) = 8.347 + 531.7 BCI	5.43	0.111
Phosphorous (P)	P (mg/dL) = 11.74 - 1596 BCI	11.73	0.017
Cholesterol (Cho)	Cho (mg/dL) = 12.73 + 9465 BCI	8.61	0.043
Triglycerides (Tri)	Tri (mg/dL) = 216.9 + 83310 BCI	10.35	0.026
Albumin (Alb)	Alb (g/dL) = 3.009 - 70.8 BCI	0.64	0.588

* BM / TL^2: Body mass (BM) divided by the square of the total length (TL^2)

Supplementary Table S3. Results of regression analysis between the Body Condition Index (BCI: BM / TL^3)* and biochemical parameters among adult capybaras (animals from human-modified and natural landscapes were all analyzed together).

**Parameters**	**Regression equation**	**R^2 (%)**	***P* value**
Fructosamine (Fru)	Fru (mmol/L) = 214,6 - 1089559 BCI	0.49	0.637
Total protein (TP)	TP (g/dL) = 7,007 - 27660 BCI	2.29	0.305
Aspartate aminotransferase (AST)	AST (U/L) = 105,8 - 2659652 BCI	7.71	0.056
Alkaline phosphatase (ALP)	ALP (U/L) = 110,5 + 2106790 BCI	0.49	0.680
Creatine kinase (CK)	CK (U/L) = 13414 - 4,16E+08 BCI	5.78	0.100
Urea (Ur)	Ur (mg/dL) = 48,05 - 535233 BCI	1.01	0.496
Calcium (Ca)	Ca (mg/dL) = 8,371 + 67297 BCI	4.57	0.144
Phosphorous (P)	P (mg/dL) = 11,85 - 208188 BCI	10.49	0.025
Cholesterol (Cho)	Cho (mg/dL) = 15,30 + 1124839 BCI	6.39	0.083
Triglycerides (Tri)	Tri (mg/dL) = - 126,3 + 7567304 BCI	4.49	0.148
Albumin (Alb)	Alb (g/dL) = 2,676 + 2318 BCI	0.04	0.898

* BM / TL^3: Body mass (BM) divided by the cube of the total length (TL^3)

Supplementary Table S4. Results of regression analysis between the Body Condition Index (BCI: resBM / TL^2)* and biochemical parameters among adult capybaras (animals from human-modified and natural landscapes were all analyzed together).

**Parameters**	**Regression equation**	**R^2 (%)**	***P* value**
Fructosamine (Fru)	Fru (mmol/L) = 182.1 - 12385 BCI	1.06	0.487
Total protein (TP)	TP (g/dL) = 6.184 - 258.3 BCI	3.35	0.213
Aspartate aminotransferase (AST)	AST (U/L) = 26.90 - 21586 BCI	8.52	0.044
Alkaline phosphatase (ALP)	ALP (U/L) = 172.5 + 9313 BCI	0.17	0.809
Creatine kinase (CK)	CK (U/L) = 1074 - 3380168 BCI	6.40	0.083
Urea (Ur)	Ur (mg/dL) = 32.16 - 4426 BCI	1.16	0.466
Calcium (Ca)	Ca (mg/dL) = 10.37 + 545.5 BCI	5.04	0.125
Phosphorous (P)	P (mg/dL) = 5.671 - 1611 BCI	10.54	0.024
Cholesterol (Cho)	Cho (mg/dL) = 48.71 + 9591 BCI	7.79	0.055
Triglycerides (Tri)	Tri (mg/dL) = 98.48 + 63581 BCI	5.31	0.115
Albumin (Alb)	Alb (g/dL) = 2.745 + 20.9 BCI	0.05	0.881

* resBM / TL^2: Residual of linear regression of body mass (resBM) divided by the square of total length (TL^2)

Supplementary Table S5. Results of regression analysis between the Body Condition Index (BCI: resBM / TL^3)* and biochemical parameters among adult capybaras (animals from human-modified and natural landscapes were all analyzed together).

**Parameters**	**Regression equation**	**R^2 (%)**	***P* value**
Fructosamine (Fru)	Fru (mmol/L) = 182.2 - 1632520 BCI	1.11	0.477
Total protein (TP)	TP (g/dL) = 6.186 - 32545 BCI	3.21	0.223
Aspartate aminotransferase (AST)	AST (U/L) = 26.99 - 2926483 BCI	9.45	0.034
Alkaline phosphatase (ALP)	ALP (U/L) = 172.6 + 1537157 BCI	0.28	0.756
Creatine kinase (CK)	CK (U/L) = 1086 - 4.62E+08 BCI	7.21	0.065
Urea (Ur)	Ur (mg/dL) = 32.18 - 589569 BCI	1.24	0.451
Calcium (Ca)	Ca (mg/dL) = 10.36 + 69404 BCI	4.92	0.130
Phosphorous (P)	P (mg/dL) = 5.685 - 202737 BCI	10.08	0.028
Cholesterol (Cho)	Cho (mg/dL) = 48.62 + 1180570 BCI	7.13	0.067
Triglycerides (Tri)	Tri (mg/dL) = 97.87 + 7824816 BCI	4.86	0.132
Albumin (Alb)	Alb (g/dL) = 2.744 + 980 BCI	0.01	0.956

* resBM / TL^3: Residual of the linear regression of the body mass (resBM) divided by the cube of the total length (TL^3)
